# A computational study on mitogenome-encoded proteins of *Pavo cristatus* and *Pavo muticus* identifies key genetic variations with functional implications

**DOI:** 10.1186/s43141-023-00534-5

**Published:** 2023-08-07

**Authors:** Shahla Yasmin, Sushant Kumar, Gajendra Kumar Azad

**Affiliations:** 1 Department of Zoology, Patna University, Patna, Bihar India; 2 Molecular Biology Laboratory, Department of Zoology, Patna University, Patna, 800005 Bihar India

**Keywords:** *Pavo muticus*, *Pavo cristatus*, Mitogenome, Protein-coding genes, Intrinsic protein disorder, Variations; Stability

## Abstract

**Background:**

The *Pavo cristatus* population, native to the Indian subcontinent, is thriving well in India. However, the *Pavo muticus* population*,* native to the tropical forests of Southeast Asia, has reduced drastically and has been categorised as an endangered group. To understand the probable genetic factors associated with the decline of *P. muticus*, we compared the mitogenome-encoded proteins (13 proteins) between these two species.

**Results:**

Our data revealed that the most frequent variant between these two species was mtND1, which had an alteration in 9.57% residues, followed by mtND5 and mtATP6. We extended our study on the rest of the proteins and observed that cytochrome c oxidase subunits 1, 2, and 3 do not have any change. The 3-dimensional structure of all 13 proteins was modeled using the Phyre2 programme. Our data show that most of the proteins are alpha helical, and the variations observed in *P. muticus* reside on the surface of the respective proteins. The effect of variation on protein function was also predicted, and our results show that amino acid substitution in mtND1 at 14 sites could be deleterious. Similarly, destabilising changes were observed in mtND1, 2, 3, 4, 5, and 6 and mtATP6–8 due to amino acid substitution in *P. muticus*. Furthermore, protein disorder scores were considerably altered in mtND1, 2, and 5 of *P. muticus*.

**Conclusions:**

The results presented here strongly suggest that variations in mitogenome-encoded proteins of *P. cristatus* and *P. muticus* may alter their structure and functions. Subsequently, these variations could alter energy production and may correlate with the decline in the population of *P. muticus*.

## Background

The Indian peafowl (*Pavo cristatus*) and green peafowl (*Pavo muticus*) are attractive birds with ornamental long tail feathers [1]. Both these species have encountered human interference, but the population of *Pavo muticus* has sharply declined in the past three decades. *Pavo muticus* had a broad distribution pattern in the 1990s and was found in the region of East and Southeast Asia [2]; however, its population has drastically declined due to several reasons including habitat destruction, poaching, and other anthropogenic activities [3–5]. Subsequently, *P. muticus* has been classified as ‘endangered’ by the International Union for the Conservation of Nature (IUCN) [3, 6]. It requires special attention from governments and local authorities for ensuring its survivability and implementation of conservation efforts. Problems of poaching (eggs, chicks, peacock, peahen, and their tail feathers) are common for both the species; however, only the *P. muticus* population has declined, indicating that apart from human interventions, there could be some genetic reasons associated with population depletion. This study was conducted with the aim to comparatively study the genomic features of these two species to determine differences between them at the genetic level. Here, we focused on comparing the mitochondrial genome (mitogenome) of these two species.

The study of mitogenome has been widely used to understand comparative and evolutionary genomics, population genetics, and phylogenetic relationships between different species [7]. The mitogenome follows maternal inheritance and demonstrates low recombination frequency. The animal mitogenome is typically circular and possesses 37 genes. Among these, 13 are protein-coding genes (PCGs) that include mtNADH dehydrogenase subunits 1–6 (mtND1–6) and 4L, cytochrome *c* oxidase subunits I–III (mtCOXI–III), ATP synthase subunits 6 and 8 (mtATP6–8), and Cytochrome b (mtCYTB). Interestingly, all these 13 proteins encoded by the mitogenome play an indispensable role in production of ATP or energy metabolism [8]. The variants of these 13 PCGs will have direct influence on the energy metabolism. Therefore, these genes are being studied to understand the role of mitogenome in adaptive evolution [9–11], such as the high-altitude adaptation in mitogenome of the Tibetan horses. It was observed that the Tibetan horses residing at higher altitudes had more nonsynonymous variants in mtND6 gene [8]. Various studies have demonstrated the variations in mitochondrial-encoded protein-coding genes (PCGs) are associated with adaptations of animals to different environmental conditions [12–16]. Likewise, in birds also, such adaptations have been reported. A study on the mitochondrial copy number of 92 avian families revealed a significant association with longevity [17]. Similarly, the mitogenomic duplications were associated with phenotypic features, and parrots with the duplicated region can live longer and show larger body mass as well as predispositions to a more active flight [18]. In Coccinellidae (ladybirds), adaptive changes in mtCOX3 were associated with metabolic differences resulting from dietary shifts [19]. In 13 crane species, the mtDNA shared a tandem duplicated region, which consists of duplicated sequence sets including mtCYTB and mtNAD6 [20] indicating the concerted evolution [20]. A study on the plum-headed parakeet (*Psittacula cyanocephala*) indicated mtNAD1 and mtNAD4L are under the strongest purifying selection, whereas mtNAD4, 5, and 6 are under the lower selection pressure, which is governing the evolution of Psittaciformes [21]. Another study demonstrated the evidence for the positive selection in the mtND2, mtND4, and mtATP6 in the high-altitude lineages of galliform birds [22]. Adaptation to different thermal environments has been correlated with the OXPHOS proteins, mtCOX and mtCYTB, encoded by the mitogenome [23, 24]. Altogether, mitogenome-encoded PCGs together with many nuclear genes may play important role in evolutionary processes [25]﻿.

This study was conducted to understand the correlation of changes in the mitogenome-encoded 13 PCGs of *P. cristatus* and *P. muticus*. Our results revealed that *P. muticus* has acquired variations in approximately 10% of its residues in mtND1 followed by mtND5 and mtATP6. Several of these variants were predicted to change the secondary structure and function of proteins. This result strongly suggests that mitochondrial PCGs might have played a critical role in the better adaptability of *P. cristatus* over *P. muticus*.

## Methods

### Sequence retrieval

Complete mitochondrial genome sequences of *P. cristatus* and P. *muticus* were obtained from the NCBI-genome database. The complete mitogenome of *P. cristatus* and *P. muticus* was reported by Zhou et al. [26] and Shen et al. [27] and has been used in this study. The accession number of mitogenome of *P. cristatus* used in this study was NC_024533.1 and NC_012897.1 for *P. muticus*, respectively*.* The protein accession number of 13 mitogenome-encoded PCGs (Table 1) was retrieved from NC_024533.1 to NC_012897.1.

**Table 1:** List of protein accession number of mitogenome-encoded proteins used in this study

S. no	Protein	*Pavo cristatus* (accession number)	*Pavo muticus* (accession number)
1	NADH dehydrogenase subunit 1	YP_009047807.1	YP_003002007.1
2	NADH dehydrogenase subunit 2	YP_009047808.1	YP_003002008.1
3	Cytochrome c oxidase subunit 1	YP_009047809.1	YP_003002009.1
4	Cytochrome c oxidase subunit 2	YP_009047810.1	YP_003002010.1
5	ATP synthase F0 subunit 8	YP_009047811.1	YP_003002011.1
6	ATP synthase F0 subunit 6	YP_009047812.1	YP_003002012.1
7	Cytochrome c oxidase subunit 3	YP_009047813.1	YP_003002013.1
8	NADH dehydrogenase subunit 3	YP_009047814.1	YP_003002014.1
9	NADH dehydrogenase subunit 4L	YP_009047815.1	YP_003002015.1
10	NADH dehydrogenase subunit 4	YP_009047816.1	YP_003002016.1
11	NADH dehydrogenase subunit 5	YP_009047817.1	YP_003002017.1
12	Cytochrome b	YP_009047818.1	YP_003002018.1
13	NADH dehydrogenase subunit 6	YP_009047819.1	YP_003002019.1

### Identification of variations between *P. cristatus* and *P. muticus* mitogenome

The multiple sequence alignment of the mitogenome-encoded 13 proteins from *P. cristatus* and *P. muticus* were performed by Clustal Omega webserver as described earlier [28, 29]*.*

### Prediction of the 3-dimensional structure of protein

We used the Phyre2 programme to predict the 3D structure of all 13 proteins encoded by the *P. cristatus* mitogenome [30]. The protein modeling by Phyre2 produces a set of potential 3D models of proteins based on alignment to known protein structures [30]. The pipeline involves the following: detecting sequence homologues, predicting secondary structure, constructing a hidden Markov model (HMM) of sequence, scanning the HMM against experimentally solved structures and constructing 3D models of protein, applying fitting procedure (cyclic coordinate descent) and a set of empirical energy terms, and modeling of amino acid side-chains based on avoidance of steric clashes [30]. This procedure can produce an accurate core of the protein within 2–4 Å root-mean-square deviation (RMSD) from the native models [30]. The structural modeling was performed using ‘normal mode’ in the Phyre2 programme. The visualisation of the predicted protein structure obtained from Phyre2 was conducted using the ChimeraX tool [31] as described earlier [32–34].

### Prediction of the effect of variations on protein intrinsic disorder, function, and stability

The PONDR-VSL2 webserver was used to predict the intrinsic disorder distribution of each residue of the protein as described earlier [35–37]. The disorder score ranges between 0 and 1, where ‘0’ represents maximum order and ‘1’ represents maximum disorder. The PROVEAN tool was used to predict the impact of variations on the protein function [38]. A PROVEAN score of − 2.5 has been considered the threshold value. The PROVEAN value equal or less than − 2.5 represents ‘deleterious variation’, while the score more than − 2.5 represents ‘neutral variation’. The I-Mutant Suite webserver [39] was used to predict the effect of variations on protein stability. This webserver predicts the differences in free energy (*ΔΔ*G) between the wild-type and mutant polypeptide sequences. The positive and negative value of *ΔΔ*G represents stabilisation and destabilisation, respectively.

## Results

### Identification of variations between the proteins encoded by the mitochondrial genome of *P. cristatus* and *P. muticus*

The polypeptide sequences of thirteen proteins encoded by the mitogenome of *P. cristatus and P*. *muticus* were extracted from the NCBI-genome database (Table 1). Each of these proteins from the two species was compared by Clustal Omega tool to identify the variations between them (listed in Table 2). Our analysis revealed that the NADH dehydrogenase subunit 1 (mtND1) has accumulated maximum variations (9.57% residues mutated), followed by NADH dehydrogenase subunit 5 (mtND5) and ATP synthase F0 subunit 6 (mtATP6) having 3.14% and 3.08% residues mutated, respectively (Table 2). Moreover, no variants were observed for cytochrome c oxidase subunits 1, 2, and 3. Furthermore, 31 sites were mutated in mtND1 followed by variations at 19 sites in mtND5 (Table 2).

**Table 2:** Summary of variations observed between *P. cristatus* and *P. muticus*. The data was obtained by comparing the mitochondrial protein sequences of *P. cristatus* and *P. muticus*

S. no	Mitochondrial genome-encoded proteins	Total protein length	Number of point mutations observed in *P. muticus* wrt *P. cristatus* (*P. cristatus* considered as wild type)	% mutant residues in *P. muticus*
1	NADH dehydrogenase subunit 1	324	31	9.57
2	NADH dehydrogenase subunit 2	346	4	1.15
3	Cytochrome c oxidase subunit 1	516	No mutation	0
4	Cytochrome c oxidase subunit 2	227	No mutation	0
5	ATP synthase F0 subunit 8	54	1	1.85
6	ATP synthase F0 subunit 6	227	7	3.08
7	Cytochrome c oxidase subunit 3	261	No mutation	0
8	NADH dehydrogenase subunit 3	116	2	1.72
9	NADH dehydrogenase subunit 4L	98	1	1.02
10	NADH dehydrogenase subunit 4	459	4	0.87
11	NADH dehydrogenase subunit 5	605	19	3.14
12	Cytochrome b	380	3	0.78
13	NADH dehydrogenase subunit 6	173	3	1.73

### In silico modeling of the mitogenome-encoded proteins of *P. cristatus*

**Fig. 1:**
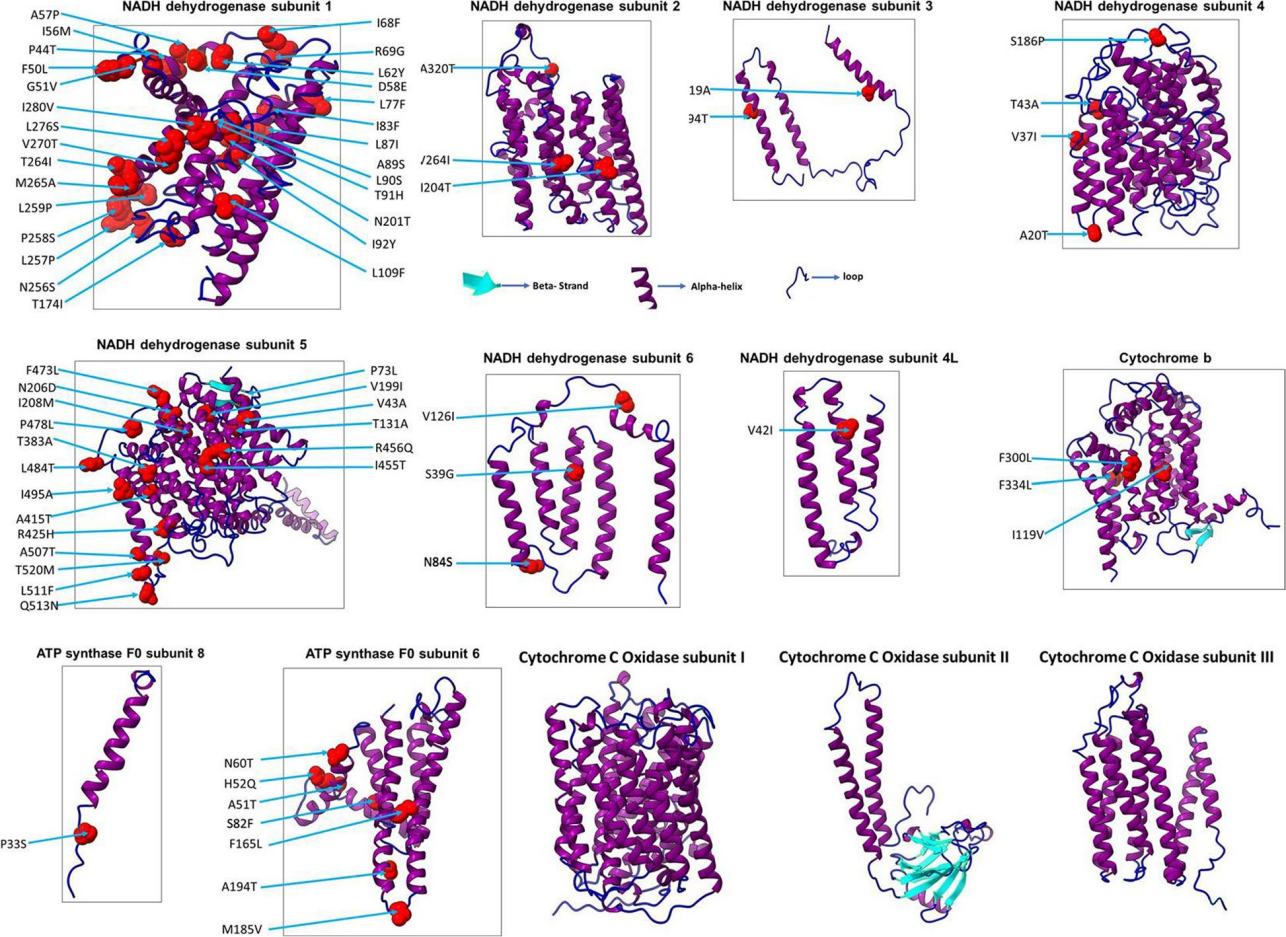
The 3D structure modeling of proteins. The 3D structure of the mitogenome-encoded protein of *P. cristatus* was modeled by the Phyre2 programme (13 structures). Amino acid substitution in the proteins of *P. muticus* is highlighted in the 3D structure of individual proteins (marked in red). The structural representation was performed by the ChimeraX tool. The structural representation demonstrates the secondary structure (alpha helix, beta-sheet, and loop) of each protein

**Fig. 2:**
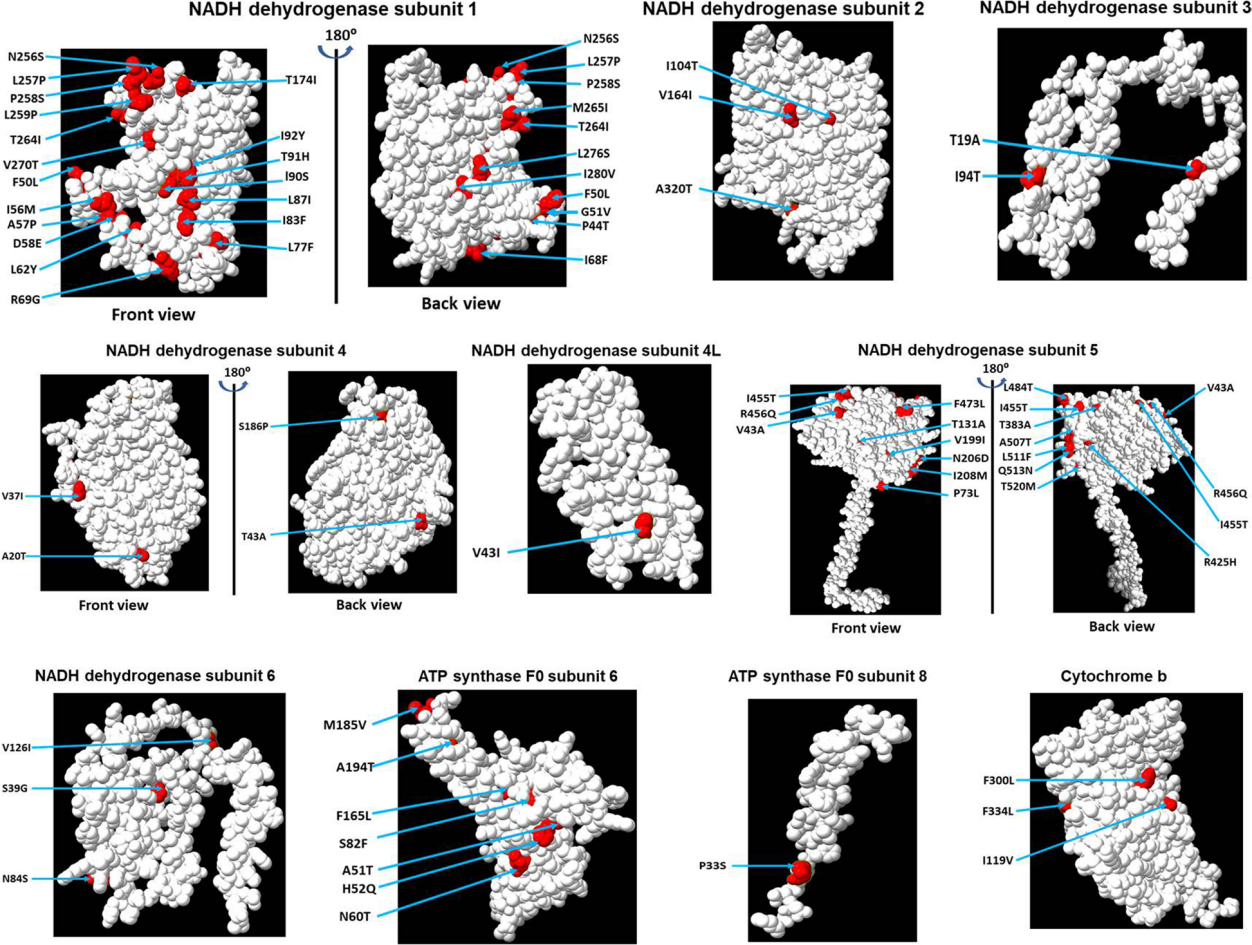
The space-filling model of proteins. The 3D structure of each mitogenome-encoded protein of *P. cristatus* was modeled by the Phyre2 programme. Amino acid substitution present in the proteins of *P. muticus* is highlighted in the 3D structure of individual proteins (marked in red). The structural representation was performed by the ChimeraX tool

The crystal structure of mitogenome-encoded proteins of *P. cristatus* and *P. muticus* is not available; therefore, we modeled them using the Phyre2 programme [30]. The 3D structure of all 13 proteins encoded by the *P. cristatus* mitogenome is shown in Fig. 1. Interestingly, it was observed that all 13 proteins are rich in alpha helix, and most of the proteins do not have a beta-sheet secondary structure (only mtND5, mtCOX2, and mtCYTB have beta-sheet) (Fig. 1). We have also highlighted the location of amino-acid substitution observed in the *P. muticus* proteins (Fig. 1, highlighted in red). Next, we analysed the location of substituted amino acids (whether they reside on protein surface or interior) by viewing the protein structure in a space-filling model. The space-filling model gives a better picture of the protein surface and shape. The amino acid residues present on the surface will interact with other proteins, while the residues present toward the interior of the protein will help in protein stability. Our data show that most of the substituted amino acid residues in *P. muticus* are present on the surface of the respective protein structure (Fig. 2). The substituted amino acids of *P. muticus* mtND1 (A89, L109, and N201) and mtND5 (A415) reside deeply buried in 3D structure (Fig. 2, these residues are not visible in space-filling model because they reside in the interior of the protein).

### The protein stability and function were also altered by amino acid substitutions

Next, we used the PROVEAN tool to analyse the effect of variations on protein function, as shown in Table 3 in terms of ‘neutral and deleterious’ (Table 3). Most of the variants were deemed ‘neutral’ because they did not have any effect on protein function; however, only three proteins showed deleterious variants such as mtND1, which had 14 variant that could be deleterious in nature. Similarly, mtND5 had 6 variants, while mtATP6 had only 1 variant that could be deleterious in nature (Table 3). Subsequently, the stability parameters were predicted by analysing the stability of proteins using the I-Mutant Suite tool (Table 3). Our data revealed that there were only a few variants that could change the stability of the protein, i.e. residues whose replacement will alter *ΔΔ*G ± 1. NADH dehydrogenase subunit 1 had 7 variants, while mtND5 had 5 variants that can destabilise protein structure (*ΔΔ*G: more than − 1) (Table 3). However, mtND 2, 3, 4, and 6 and mtATP6–8 had single destabilising variant (Table 3).

**Table 3:** Stability predictions

Sl. no	Amino acid in *P. cristatus*	Amino acid position	Amino acid in *P. muticus*	PROVEAN score/prediction	Stability prediction
NADH dehydrogenase subunit 1					
1	N	8	S	− 0.255/neutral	− 0.29
2	V	17	A	− 2.865/deleterious	− 1.78
3	P	44	T	− 7.097/deleterious	− 1.4
4	F	50	L	− 2.262/neutral	− 1.04
5	G	51	V	− 7.989/deleterious	− 0.46
6	I	56	M	− 1.816/neutral	− 1.61
7	A	57	P	− 4.404/deleterious	− 0.2
8	D	58	E	− 3.570/deleterious	− 0.22
9	L	62	Y	− 4.521/deleterious	− 1.57
10	I	68	F	− 3.299/deleterious	− 1.64
11	R	69	G	− 5.591/deleterious	− 1.49
12	L	77	F	− 3.584/deleterious	− 1.2
13	I	83	F	− 1.851/neutral	− 1.15
14	L	87	I	− 0.161/neutral	− 0.77
15	A	89	S	− 2.170/neutral	− 0.46
16	L	90	S	− 4.954/deleterious	− 1.41
17	T	91	H	− 2.878/deleterious	− 0.87
18	I	92	Y	− 3.333/deleterious	− 1.10
19	L	109	F	− 2.528/deleterious	− 0.84
20	T	174	I	− 0.630/neutral	0.08
21	N	201	T	− 5.752/deleterious	0.13
22	N	256	S	− 0.398/neutral	− 0.40
23	L	257	P	− 0.131/neutral	− 1.13
24	P	258	S	− 0.933/neutral	− 1.28
25	L	259	P	4.800/neutral	− 1.32
26	T	264	I	1.344/neutral	− 0.18
27	M	265	A	0.194/neutral	− 0.78
28	V	270	T	2.094/neutral	− 1.37
29	L	276	S	− 1.378/neutral	− 2.19
30	I	280	V	0.215/neutral	− 0.76
31	I	324	A	0.206/neutral	− 2.36
NADH dehydrogenase subunit 2					
1	I	104	T	− 1.913/neutral	− 1.81
2	V	164	I	0.755/neutral	− 0.64
3	A	320	T	0.583/neutral	− 0.57
NADH dehydrogenase subunit 3					
1	T	19	A	0.732/neutral	− 0.67
2	I	94	T	− 1.087/neutral	− 1.38
NADH dehydrogenase subunit 4					
1	A	20	T	0.133/neutral	− 0.68
2	V	37	I	0.167/neutral	− 0.57
3	T	43	A	− 0.329/neutral	− 1.39
4	S	186	P	− 0.368/neutral	− 0.07
NADH dehydrogenase subunit 5					
1	V	43	A	− 0.355/neutral	− 1.41
2	P	73	L	0.706/neutral	− 0.29
3	T	131	A	− 0.826/neutral	− 0.87
4	V	199	I	0.746/neutral	0.25
5	N	206	D	− 0.598/neutral	0.06
6	I	208	M	0.409/neutral	− 1.48
7	T	383	A	− 3.580/deleterious	− 1.22
8	A	415	T	− 3.644/deleterious	− 0.47
9	R	425	H	− 4.584/deleterious	− 0.94
10	I	455	T	2.178/neutral	− 2.18
11	R	456	Q	− 3.629/deleterious	− 0.90
12	F	473	L	1.861/neutral	− 0.85
13	P	478	L	− 6.847/deleterious	− 0.34
14	L	484	T	0.378/neutral	− 1.62
15	I	495	A	− 3.218/deleterious	− 1.85
16	A	507	T	0.165/neutral	− 0.49
17	L	511	F	0.107/neutral	− 0.73
18	Q	513	N	− 1.308/neutral	− 0.57
19	T	520	M	− 0.172/neutral	− 0.29
NADH dehydrogenase subunit 6					
1	S	39	G	5.647/neutral	− 0.91
2	N	84	S	− 0.765/neutral	− 0.26
3	V	126	I	− 0.381/neutral	− 1.14
NADH dehydrogenase subunit 4L					
1	V	42	I	0.557/neutral	− 0.72
2	I	119	V	− 0.370/neutral	− 0.39
3	F	300	L	2.338/neutral	− 1.10
4	F	334	L	1.491/neutral	− 1.09
ATP synthase F0 subunit 6					
1	A	51	T	0.285/neutral	− 0.58
2	H	52	Q	− 0.740/neutral	− 0.09
3	N	60	T	1.016/neutral	0.56
4	S	82	F	− 3.746/deleterious	0.05
5	F	165	L	3.582/neutral	− 1.00
6	M	185	V	− 0.731/neutral	− 0.40
7	A	194	T	− 0.854/neutral	− 0.31
ATP synthase F0 subunit 8					
1	P	33	S	− 1.017/neutral	− 1.68

### The variations in amino acid alter the protein intrinsic disorder parameters

**Fig. 3:**
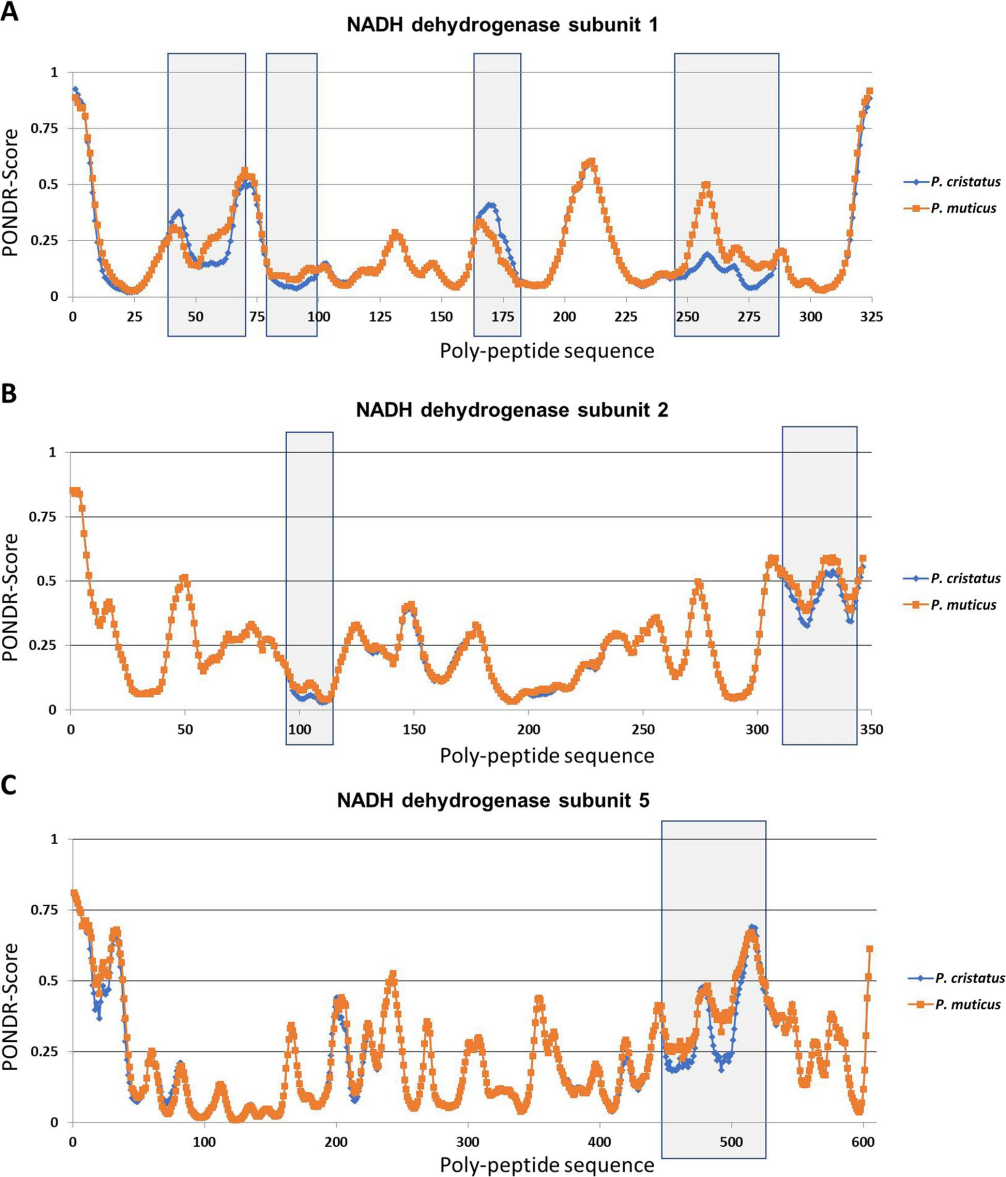
Analysis of intrinsic disorder score among the proteins of *P. cristatus* and *P. muticus*. **A** Comparison of the intrinsic disorder predisposition of mtND1, **B** mtND2, and **C** mtND5. The threshold value is depicted at 0.5; the scores > 0.5 are considered as disordered. Grey box highlights the area of the protein that exhibited considerable change in the intrinsic disorder score﻿

The prediction of intrinsic disorder parameters of each polypeptide was predicted using the PONDR-VSL2 tool. Our data demonstrate that out of 13 proteins analysed here, 10 of them do not show any considerable change in protein disorder score (data not shown). The rest of the 3 proteins mtND1, 2, and 5 exhibited changes in the protein disorder score (Fig. 3). The detailed analysis revealed that mtND1 has acquired changes in intrinsic disorder scores (Fig. 3A, location is marked by grey rectangular box). Similar changes were observed for mtND2 and mtND5 (Fig. 3B and C, location is marked by grey rectangular box). Altogether, our intrinsic disorder prediction revealed that variation in amino acids was translated to the alteration in disorder parameters among the mitogenome-encoded proteins of *P. cristatus* and* P*. *muticus*.

## Discussion

*P. cristatus* population is thriving very well in India, but the *P. muticus* population has shrunken drastically, and it has been categorised into an endangered group. Therefore, to understand the probable genetic factors associated with the decline of *P. muticus*, we compared the mitogenome-encoded proteins between these two species. Several studies have reported that the variations in mtND1/2/3/4/4L/5/6 lead to different genetic diseases in humans [40–43], such as Leber hereditary optic neuropathy, Leigh syndrome, and myocardial mitochondrial disease, indicating that these proteins play a critical role in maintaining the normal homeostasis of an organism. In this study, the most frequent variations were observed for mtND1 in *P. muticus* that had alteration in 9.57% of residues compared to the same protein in *P. cristatus*. The mtND1 gene is involved in the first step of the electron transport chain of oxidative phosphorylation (OXPHOS). The alteration of the electron transport components by variations in mtDNA may compromise the normal electron flow, leading to inefficient energy production. It is possible that alteration in 9.57% residues of mtND1 in *P. muticus* causes a defect in energy production as compared to *P. cristatus*, leading to a decline of *P. muticus* population. In human cancers, three variants, Y30H, Y43C, A64V, and few others, have been observed that can alter the structure and function of the mtND1 protein [44, 45]. In this study, we also observed variations at 9 residues in *P. muticus* mtND1 between amino acid residues 30–70, indicating that these variations may cause alteration in the structure and function of the mtND1 protein. The mtND5 had 19 variations in *P. muticus* in comparison to the same protein of *P. cristatus*. Interestingly, mtND5 variants have been extensively studied and found to cause several mitochondrial diseases [46] including LHON [47], adult encephalopathy [48], MELAS [49], and Leigh syndrome [50]. These data indicate that mtND5 is a very important player of the mitochondria, and the 19 variations observed in this protein of *P. muticus* may cause defect in mtND5 functions.

The mtATP6 and mtATP8 are proteins that function as part of the F0 component (proton pump) of the F0F1 complex. These two proteins are directly involved in proton transport, which has physiological implications. Our data show that MtATP6 of *P. muticus* has attained variations at 6 positions (compared to *P. cristatus*). Several studies have revealed the biochemical consequences of amino acid replacement of mtATP6 and indicated that they may alter mitochondrial ATP production, ranging from 30 to > 90% [51–54]. In another study, the mtATP6 of *Champsocephalus gunnari* (ice fish) was compared with the same protein from a few vertebrate species, and their data revealed that the variations observed between them could affect the structure and function of the protein leading to the unique physiology of the ice fish [55]. A similar effect might occur due to the variations observed at 6 positions in mtATP6 of *P. muticus*. We extended our study on the rest of the proteins encoded by the mitogenome and observed that cytochrome c oxidase subunits 1, 2, and 3 do not have any change between *P. muticus* and *P. cristatus*, indicating that these proteins are highly conserved. The mtND2, 3, 4, 4L, and 6 and cytochrome b have only few variants between these two species and could have structural changes. It has been observed that mtND6 shows specific changes that correlate with high-altitude environment adaptability in the plateau horse [56]. There are evidences indicating the role of mtND2 and 4 and mtATP6 in high-altitude adaptation among galliform birds [22]. Furthermore, most of the variants reported in this study had no effect on protein function (neutral); however, only three proteins showed deleterious variations, including mtND1, mtND5, and mtATP6. A similar comparative study on the mitogenome of shallow-sea and deep-sea starfish revealed the variation specifically in mtATP8 and MtND2 and 5 required for deep‐sea environment adaptations [57]. Moreover, the role of mitogenome-encoded PCGs provided the genetic basis of deep‐sea hydrothermal vent adaptation in the shrimp [58]. In avians, the mitochondrial copy number has been associated with longevity [17]. In parrots, the mitogenomic duplications have been found to be associated with enhanced longevity [18]. Similarly, in ladybirds, adaptive changes in mitogenome were associated with metabolic differences resulting from dietary shifts [19].

In this study, we have also observed multiple variations in mitogenome of *P. muticus* and *P. cristatus* that may affect adaptations and survivability. Importantly, the population of *P. muticus* has drastically declined due to habitat destruction, poaching, and other anthropogenic activities. However, our study also supposes that apart from these anthropogenic activities, the variations observed in the mitogenome could also add to the susceptibility of *P. muticus* to the changing environmental conditions.

## Conclusions

The observations reported in this study support the fact that the mitogenome-encoded proteins in *P. muticus* have attained variations that could alter energy production and may correlate with the decline in the population of *P. muticus*. However, this needs to be tested by in vivo experiments using proteins from these two species. Altogether, we provide probable consequences of variations in the mitogenome of *P. muticus* and *P. cristatus* that could have a survivability effect.

## Data Availability

Not applicable.
